# Thin-layer liquid-based cervical cytology and PCR for detecting and typing human papillomavirus DNA in Flemish women

**DOI:** 10.1038/sj.bjc.6600756

**Published:** 2003-02-18

**Authors:** C E Depuydt, A J Vereecken, G M Salembier, A S Vanbrabant, L A Boels, E van Herck, M Arbyn, K Segers, J J Bogers

**Affiliations:** 1 Laboratory for Clinical Pathology (Labo RIATOL), Amerikalei 62-64, B-2000 Antwerp, Belgium; 2 European Network for Cervical Cancer Screening, Scientific Institute of Public Health, Juliette Wytsmanstreet 14, B-1050 Brussels, Belgium; 3 Laboratory of Pathology, University Hospital Antwerp, B-2650 Edegem, Belgium

**Keywords:** human papillomavirus (HPV), cervical cancer screening, Belgium, Flanders

## Abstract

The objective of this study was to document the occurrence and to correlate the prevalence of different human papillomavirus (HPV) types with the cytological results on simultaneously performed thin-layer preparations in a large population of Flemish women. During 1 year, 69 290 thin-layer preparations were interpreted using the Bethesda classification system. Using an algorithm for HPV testing based on consensus primers and type-specific PCRs in combination with liquid-based cytology, we determined the occurrence and distribution of 14 different oncogenic HPV types (16, 18, 31, 33, 35, 39, 45, 51, 52, 56, 58, 59, 66 and 68). Reflex HPV testing was performed on cytologically abnormal samples and on an age matched randomly selected control group with normal cervical cytology (*n*=1351). Correlation between cytology, age and prevalence for the 14 different high-risk HPV types is given. There is a significant increase in predominance of high-risk HPV types, with increasing abnormal cytology. Coinfection with multiple HPV types also increased with cytological abnormalities, and was highest in HSIL (16.7%). In Flanders, HSIL was most often associated with HPV types 16, 33, 35, 31, 18 and 51. Using thin-layer liquid-based cytology and PCR to detect HPV, it is feasible to screen large numbers of women.

During recent years, there has been increasing evidence implicating infection with high-risk human papillomavirus (HPV) as a causal factor in the development of cervical intraepithelial neoplasia and invasive carcinoma (Kiviat and Koutsky, 1993; Schiffman *et al*, 1993; IARC Working Group, 1995; Bosch *et al*, 2002). The presence of HPV in virtually all cervical cancers implies the highest worldwide attributable fraction so far reported for a specific cause of any major human cancer (Walboomers *et al*, 1999). Worldwide cervical cancer remains the second most common cancer in women (Parkin *et al*, 2001). In Belgium cervical cancer ranks fifth after breast, colon, ovarium and rectum cancer (Arbyn and Van Oyen, 1999; National Cancer Register, 2000).

More than 40 genital HPV types have been shown to infect the genital mucosa and have been characterised (IARC Working Group, 1995). Recent studies have shown that in addition to HPV 16 and 18, HPV types 31, 33, 35, 45, 51, 52, 58 and 59 should be considered as human carcinogens (Bosch *et al*, 2002). The potential role of HPV testing in cervical cancer screening programmes has been under consideration for several years (Cuzick *et al*, 1994; Jenkins *et al*, 1996) and is felt to be promising for a triage of women with atypical squamous cells of undetermined significance (Solomon *et al*, 2001; Arbyn and Van Ranst, 2002). Identification of high-risk HPV genotypes may permit selection of patients who are at increased risk for disease (Cuzick *et al*, 1995). For women with low-grade squamous intraepithelial lesions there is only a limited potential for triage with HPV DNA testing because a very high percentage of these women were shown to be HPV positive (ALTS group, 2000; Stoler, 2001). However, detection of type-specific HPV DNA in abnormal smears could be used as a more specific predictor of high-grade cervical intraepithelial neoplasia (Cuzick *et al*, 1994).

We used the MY09/MY11 L1 consensus primer-based PCR (Bauer *et al*, 1991), in combination with 14 type-specific PCRs (Walboomers *et al*, 1999), to determine the prevalence of different oncogenic HPV types in a group of women with normal and abnormal cervix cytology. Owing to its complex politico-administrative structure, Belgium can be divided into three geographically separated regions. The Flemish region, with a female population of 3.0 million, the Walloon region with 1.7 million women and the Capital region of Brussels with 0.5 million women. A programme for organised screening, based on the European guidelines, only exists in the Flemish community (Arbyn and Van Oyen, 2000). The aim of the study was to document the occurrence and distribution of HPV types in a population of Flemish women, and to correlate the prevalence of different HPV types with the cytological results on simultaneously performed thin-layer preparations.

## MATERIALS AND METHODS

### Sample processing and cytological procedure

For thin-layer liquid-based cytology preparations (LBP), cervical samples were collected using the Cervex-Brush® (Rovers, Oss, The Netherlands). After collection, brush heads were transferred directly into alcohol-based preservative (AutoCyte®, Tripath Imaging Inc, Burlington, NC, USA), and the vials were transported to the laboratory. The LBP were made with the fully robotic AutoCyte® PREP System (AutoCyte®, Tripath Imaging Inc, Burlington, NC, USA) (Vassilakos *et al*, 1999), and were prepared as described elsewhere (Vassilakos *et al*, 1998). All slides were manually screened by cytotechnologists who are intensively trained in the evaluation of thin-layer slides. Five percent of all LBPs (quality control), as well as all positive and suspicious cases were reviewed by senior cytotechnologists and forwarded for final diagnosis to one of the pathologists (KS, JJB), who were unaware of the subject's HPV DNA status. The cytological results were classified according to the Bethesda system (Kurman and Solomon, 1996), using the classes within normal limits (WNL), atypical squamous cells of undetermined significance (ASCUS), low-grade squamous intraepithelial lesions (LSIL) and high-grade squamous intraepithelial lesions (HSIL).

### Screening procedure and patients

Between July 2000 and June 2001, the Laboratory for Clinical Pathology (Labo RIATOL, Antwerp, Belgium) received 69 290 cervical samples for cytological evaluation from women that were taken by general practitioners and gynaecologists in Flanders (Belgium).

HPV DNA reflex testing was performed on all cytologically abnormal samples. For cytological normal samples, HPV DNA testing was performed only on explicit request of the clinician (WNLr), and on a randomly selected representative control group of 100 samples with normal cervical cytology (WNLc) of women with the same age distribution as the total group.

Study-specific patient identification codes were assigned and transmitted in such a manner that patient confidentiality was preserved.

### Isolation of DNA from cervical cells

After thin-layers were made, 400 *μ*l of the remaining cells suspension was transferred to an eppendorf tube and cells were pelleted by centrifugation. The supernatant was discarded and the pellet resuspended in 50 *μ*l digestion solution (10 mM Tris, 1 mM EDTA, 200 *μ*g ml^−1^ Proteinase K) and digested for 3 h at 55°C. The digestion was followed by a 10 min incubation at 95°C to inactivate the proteinase K. The DNA extracts were stored at −20°C until PCR was performed.

### Positive and negative controls for PCR analysis

Several precautions were taken to prevent false positive results. Different steps such as DNA extraction, sample preparation, amplification and post-PCR were performed in strictly separated rooms. Aerosol-resistant pipette tips were used for all handling of liquids. In all runs, distilled water samples were included as negative PCR controls. As positive control samples, HPV-containing cell lines SiHa (ATCC, HTB-35), containing 1 copy of HPV-16/cell, and Hela (ATCC, CCL-2) containing 10–50 HPV-18 copies/cell were used.

### HPV detection and typing by PCR

PCR using consensus primers MY09/MY11 allowed the production of 450 bp fragments in the L1 region (Bauer *et al*, 1991). Specimens positive with the consensus PCR were assayed with E7 type-specific primers for high-risk HPV types 16, 18, 31, 33, 35, 39, 45, 51, 52, 56, 58, 59, 66 and 68 (van den Brule *et al*, 1990), and were done in sequence (Walboomers *et al*, 1999). We tested for HPV types 45, 52, 56, 58, 59 and 68 only in patients who where HPV type 16, 18, 31, 33, 35, 39, 51 and 66 negative. Cases in which general primer PCR was positive, but negative in the type-specific PCRs, were considered to contain presently unidentified HPV genotypes and were denoted HPV X. Samples negative for the consensus PCR were subjected to a control PCR for *β*-globin with GH20 and PC04 primers (Manos *et al*, 1989) and type-specific PCRs for HPV types 18, 35, 39, 51 and 66. A measure of 10 *μ*l of each PCR product was electrophoresed in a 3% agarose gel and stained with ethidium bromide. Three experienced investigators interpreted each result, and discrepancies were resolved by consensus.

### Statistical analysis

Comparisons of means was studied by analysis of variance (ANOVA), followed by Student–Newman–Keuls test for all pairwise comparisons. The *χ*^2^ statistics for trend was used to verify the existence of a trend across ordered groups (such as increase in HPV positivity) according to the degree of cytological abnormality. Statistical tests were considered significant at *P*<0.05. Statistical analysis was performed using the MedCalc® program (MedCalc Software, Mariakerke, Belgium) (Schoonjans *et al*, 1995).

## RESULTS

From a total of 69 290 samples, 19 (0.03%) were inadequate, 67 474 (97.38%) had normal cytology, 992 (1.43%) were classified as ASCUS, 641 (0.93%) as LSIL and 164 (0.24%) as HSIL. In total, 1797 (2.59%) samples were classified as abnormal (Table 1

**Table 1:** Baseline characteristics of the different cytologic groups according to the Bethesda classification

	**Whole group**				**HPV-tested group**			
**Bethesda**	***n***	**Mean age**	**s.d.**	**Range**	***n***	**Mean age**	**s.d.**	**Range**
WNL	67 474	40.4	13.1	13–95	100^c^, 193^r^	40.1	13.1	17–85
ASCUS	992	38.0	13.0	16–87	552	37.2	12.4	16–78
LSIL	641	32.9	11.0	16–78	369	32.9	10.9	17–74
HSIL	164	39.3	11.5	19–79	137	39.7	11.8	21–77
								
Total	69 290*	40.3	13.1	13–95	1351	40.3	12.3	16–85

WNL=within normal limits;

c =control group;

r =tested on request of clinician;

ASCUS=atypical squamous cells of undetermined significance; LSIL=low-grade squamous intraepithelial lesions; HSIL=high-grade squamous intraepithelial lesions; s.d.=standard deviation;

* =19 samples were inadequate.

). The mean age of the total screened population was 40.3 years. Surprisingly, the mean age of the LSIL group (32.9 years) was significantly lower (*P*<0.05) compared to all other groups.

From the total group of abnormal samples, 129 samples were not tested because the clinician did not want HPV reflex testing on abnormal samples, and 610 samples of women who were tested shortly before were excluded from the study population. Ultimately 1058 abnormal samples were included, 552 ASCUS, 369 LSIL and 137 HSIL cases. Also, 193 samples with normal cytology were tested for HPV DNA on explicit demand of the clinician (Table 1). Also for the HPV DNA-tested samples, the mean age of the LSIL group (32.9 years) was significantly lower (*P&*lt;0.05) compared to all other groups. There was no significant difference in age between the cytologic groups tested for HPV and cytologic groups of the whole group (*P*>0.05).

### HPV DNA positivity

**Figure 1:**
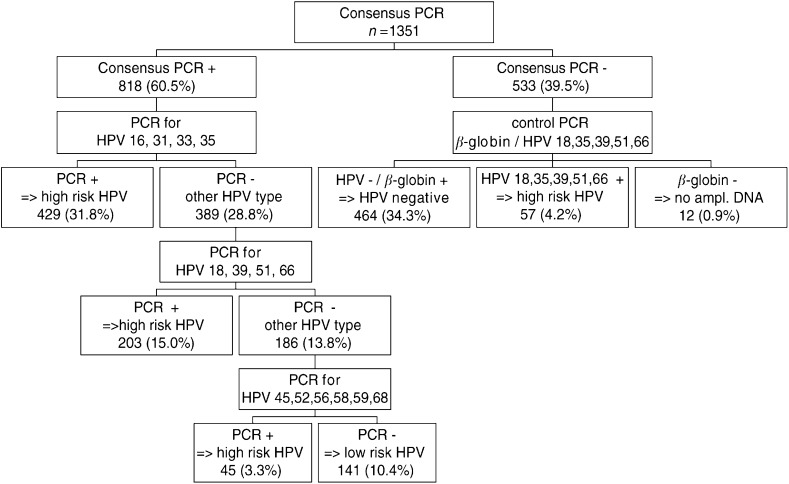
Algorithm for HPV PCR testing.

Using the consensus PCR as a first step for triage, 818 samples revealed to be positive for HPV DNA (Figure 1

). In 12 samples from the 533 samples that tested negative for the consensus PCR, no *β*-globin could be amplified, and 57 tested positive for HPV types 18, 35, 39, 51 or 66. HPV51 was the most frequent type that was not detected by the consensus PCR (71.9%), followed by HPV39 (12.3%), HPV18 and 35 (7.0%), and HPV66 (3.5%). When we consider the number of samples tested in each cytological diagnosis group, the number of consensus PCR-negative samples, but containing HPV types 18, 35, 39, 51, 66, was most frequent in the LSIL (8.9%) and HSIL (4.5%) groups (Table 2

**Table 2:** Overall HPV prevalence and type distribution detected by type-specific PCRs of consensus PCR-negative samples according to cytological diagnosis

**HPV type**	**WNLc**	**WNLr**	**ASCUS**	**LSIL**	**HSIL**
18	—	—	1	2	—
18,39	—	—	—	—	1
35	—	1	—	2	1
39	—	2	2	2	—
51	1	1	10	26	3
66	—	—	—	1	1
					
Total HPV +	1	4	13	33	6
Tested/group	100	187	549	369	134
Per cent +/group	1.0	2.1	2.4	8.9	4.5

For abbreviations see footnote in Table 1.

). Finally, taking into consideration samples positive with type-specific PCRs but consensus PCR negative, overall 464/533 samples tested negative for HPV (Figure 1).

Typing of consensus positive samples with a first multiplex PCR detecting HPV types 16, 31, 33 and 35 revealed that more than half of the tested samples (429 out of 818; 52.4%) were positive for one of these HPV types. Subsequent typing of the remaining 389 samples with multiplex PCRs for types 18, 39, 51 and 66 showed that 52.2% (203 out of 389) of samples contained these oncogenic HPV types. From the remaining 186 untyped samples, only 45 were positive when tested with type-specific PCRs for HPV types 45, 52, 56, 58, 59 and 68. Ultimately, 141 samples were negative for all type-specific PCRs and were considered to contain presently unidentified HPV genotypes of unknown malignant potential (HPV X).

The overall HPV prevalence and type distribution according to cytological diagnosis is given in Table 3

**Table 3:** HPV prevalence and type distribution according to cytological diagnosis

**HPV type**	**WNLc**	**WNLr**	**ASCUS**	**LSIL**	**HSIL**
16	1	9	61	65	55
16,31	—	—	—	2	—
16,31,35	1	—	3	2	2
16,33	—	—	3	2	5
16,35	1	—	4	4	5
18	1	3	30	20	5
18,39	—	1	2	—	2
18,66	—	—	—	2	—
31	—	—	1	6	—
31,33,35	—	—	1	—	—
31,35	—	—	14	29	7
33	—	5	32	39	22
33,35	—	—	1	2	1
35	—	5	19	15	9
39	1	7	25	32	2
45	—	—	1	1	2
51	2	3	28	45	7
52	—	1	9	3	4
56	—	—	5	15	—
58	—	—	3	—	—
59	—	—	1	—	—
66	—	—	9	28	1
68	—	—	—	—	—
X	14	14	70	40	3
					
Σ 16	3	9	71	75	67
Σ 18	1	4	32	22	7
Σ 31	1	—	19	39	9
Σ 33	—	5	37	43	28
Σ 35	2	5	42	52	24
Σ 39	1	8	27	32	4
Σ 66	—	—	9	30	1
					
Total HPV +	21	48	322	352	132
Single type	19	47	294	309	112
Multiple types	2	1	28	43	22
High-risk type(s)	7	34	252	312	129
					
Total tested/group	100	187	549	369	134

Σ=total number of samples positive for the specified HPV type; X=HPV types different from HPV 16, 18, 31, 33, 35, 39, 45, 51, 52, 56, 58, 59, 66 and 68.

. The 12 samples that tested negative for *β*-globin DNA were excluded from further analysis. Using HPV consensus L1 and E7 type-specific PCRs, the total number of HPV-positive samples was 875 out of 1339 (65.4%). Overall, HPV was detected in 21% of the samples of the WNLc group, in 25.7% of the WNLr group, in 58.7% of the ASCUS group, in 95.4% of the LSIL group and in 98.5% of the samples in the HSIL group (*χ*^2^ for linear trend=440, *P*<0.0001). The percentage of HPV-positive samples infected with only one HPV type in the WNLc and the WNLr group was 19.0 and 25.1%, respectively, and was not significantly different (*P*>0.2). There was a significant increase in samples with single HPV infection with increasing abnormal cytology (*χ*^2^ for linear trend=289, *P*<0.0001). The percentage of samples infected with multiple HPV types was low in the control group (2.0%) and did not differ significantly (*P*>0.9) from the percentage found in the WNLr group (0.5%). There was a significant increase in samples infected with multiple HPV types with increasing abnormal cytology as compared to the WNLr group (*χ*^2^ for linear trend=48, *P*<0.001). A linear trend was also found in samples infected with high-risk HPV types with increasing abnormal cytology (*χ*^2^ for linear trend=78, *P*<0.001).

### Distribution of HPV types according to cytology

In the WNLc control group only a limited number of oncogenic HPV types were detected, namely HPV 16, 18, 31, 35, 39 and 51 (Table 3). The HPV types in the WNLr group were very similar, with only additional HPV types 33 and 52 being detected. The greatest HPV-type heterogeneity was seen in the ASCUS group, where almost all oncogenic types were detected except for HPV68. HPV types 58 and 59 were only detected in the ASCUS group. Also in the group with low-grade lesions, there was a great HPV-type heterogeneity. The type spectrum in the high-grade lesions is more restricted and is comparable with the types detected in the WNLr/WNLc groups. In the HSIL group, HPV types 16, 18, 31, 33, 35, 39, 45, 51 and 52 accounted for almost 97% of all types found. From the 818 consensus PCR-positive samples, 141 were negative for all type-specific PCRs and were considered to contain presently unidentified HPV genotypes of unknown risk potential (HPV X).

**Figure 2:**
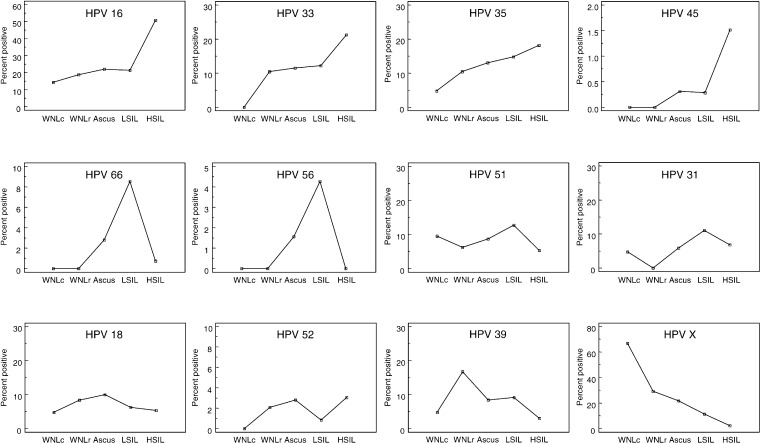
Prevalence for each HPV type according to cytological diagnosis.

The prevalence for each individual HPV type as a function of increasing abnormal cytology is shown in Figure 2

. For HPV types 16, 33, 35 and 45 there was an increasing prevalence with increasing abnormal cytology, with the highest prevalence in the HSIL group. For HPV types 66, 56, 51 and 31, the highest prevalence was observed in the LSIL group. The highest prevalence for HPV18 was seen in the ASCUS group, for HPV type 52 in both the ASCUS and HSIL groups and for HPV39 and HPVX in the WNLr and WNLc groups, respectively. The prevalence of unidentified HPV types decreased with increasing abnormal cytology. Patients with ASCUS were younger than patients with LSIL for HPV types 16, 31, 35, 51 and 56. For HPV types 18, 33, 39, 45, 52 and 66 patients with ASCUS were older than patients with LSIL.

### Age and HPV prevalence

The mean age of the different cytologic groups according to HPV DNA positivity is given in Table 4

**Table 4:** Mean age of the different cytologic groups according to HPV DNA positivity (HPV DNA-tested group)

**Bethesda**	**HPV**	***n***	**Mean age**	**s.d.**	**Range**	**Different (*P*<0.05) from**
WNLc	−	79	41.2	13.4	17–74	LSIL
	+	21	36.1^NS^	11.0	20–55	
WNLr	−	139	41.3	12.0	17–79	LSIL
	+	48	37.1^a^	13.5	18–85	LSIL
ASCUS	−	227	41.1	12.4	18–78	LSIL
	+	322	34.4^b^	11.7	16–75	LSIL*, HSIL
LSIL	−	17	33.7	11.5	17–57	WNLc, WNLr, ASCUS, HSIL
	+	352	32.8 NS	10.9	17–74	WNLr, ASCUS*, HSIL
HSIL	−	2	55.0	4.2	52–58	LSIL
	+	132	39.4 NS	11.8	19–77	ASCUS, LSIL
Total	−	464	41.0	12.5	17–79	
	+	875	34.7^b^	11.7	16–85	

a Significantly younger than the HPV-negative samples (*P*<0.04).

b Significantly younger than HPV-negative samples (*P*<0.0001).

NS =not significantly younger than HPV-negative samples (*P*>0.05).

* *P*<0.07. For other abbreviations see Table 1.

. In the WNLr and ASCUS groups, HPV-positive patients were significantly younger than HPV-negative patients (*P*<0.05). Whereas in the WNLc, LSIL and HSIL groups no significant difference in age between HPV-positive and HPV-negative patients was observed. Patients with LSIL smears were always the youngest irrespective of their HPV status.

## DISCUSSION

Each year approximately 1 200 000 cervical smears are taken in Belgium, of which 660 000 come from women living in Flanders (Arbyn and Van Oyen, 1999). Between July 2000 and June 2001, 69290 thin-layer cytology preparations were processed in our laboratory, representing almost 11% of all smears taken in Flanders during that period.

Using an algorithm for HPV testing based on consensus primers and type-specific PCRs in combination with liquid-based cytology, we determined the occurrence and distribution of 14 different oncogenic HPV types in a population of Flemish women.

### Methodological considerations

#### Thin-layer liquid-based cervical cytology

One advantage of using liquid cytology for the collection of cervical specimens is that multiple diagnostic tests can be performed on a single sample avoiding recall of women for additional testing. The technique can be easily automated and combined with HPV testing on a large number of samples. For samples diagnosed as ASCUS, LSIL or HSIL, HPV testing can be performed on the remaining stored liquid cytology specimen without the cost of a clinical follow-up visit. Furthermore, only in a small number of samples *β*-globin DNA could not be detected. Therefore, the CytoRich fixative provides an excellent preservation of the samples making a simple and cheap DNA extraction possible. Liquid-based cytology improves the overall adequacy of the cell sampling, smearing and staining, as seen by the low number of samples judged as inadequate (0.03%) or interpreted as ASCUS (1.43%). Overall, 2.6% of the smears were classified as abnormal. This is within the range observed in other Flemish cytological laboratories (Arbyn *et al*, 1999). Finally, supplementing HPV detection to liquid-based cytology in a single combined test makes available an indicator for the quality assurance of abnormal cervical cytology. Our cytological diagnostic accuracy appears consistent with the HPV detection, since a high percentage (76.6%) of abnormal samples are HPV positive.

#### HPV detection

As previous studies have shown, the MY09/11 primers preferentially amplify certain HPV types (Walboomers *et al*, 1999; Gravitt *et al*, 2000), therefore all consensus negative samples were subjected to specific PCRs for types 18, 35, 39, 51 and 66. Not unexpectedly HPV51 was the most frequent type that was not detected by the consensus PCR, followed by HPV39 and 35. It has been shown that the addition of an extra sequence-specific oligonucleotide (HMB01) directed to the minus strand of HPV type 51 facilitates the amplification of this important cancer-associated type of HPV (Hildesheim *et al*, 1994). The MY09/11 PCR targets a 450 bp fragment within the HPV L1 open reading frame. Failure to detect HPV DNA may have been because of the lack of specificity for certain HPV types (Gravitt *et al*, 2000), such as HPV51, 35 and 39. A second possibility is that the MY09/11 primers failed to amplify HPV DNA because of the loss of the HPV L1 open reading frame due to integration of HPV DNA into the host genome (Walboomers *et al*, 1999). This is particularly true for HPV18. Previous studies have shown that HPV18 is more often disrupted in the L1 region than other HPV types (Berumen *et al*, 1994; Walboomers *et al*, 1999).

#### HPV-type prevalence

The type spectrum in the high-grade lesions is much more restricted compared to ASCUS and LSIL, with HPV types 16, 18, 31, 33, 35, 39, 45, 51, 52, 58 and 59 accounting for nearly 97% of HPV types detected in HSIL. This is in agreement with other studies (Bosch *et al*, 2002), where the HPV DNA prevalence in HSIL using PCR-based methods varies from 75 to 100%.

In Flanders, as in the rest of the world (Bosch *et al*, 2002), HPV16 is the most common virus at the cervix regardless of the cytological diagnosis.

The overall HPV-type distribution in HSIL was similar to that in squamous cell carcinoma, but type distribution was dissimilar in one aspect. HPV18 was not the first or second most frequent high-risk type but rather the fourth or fifth. This is in accordance with studies from Mexico and Canada using the same MY09/11 PCR (Torroella-Kouri *et al*, 1998; Feoli-Fonseca *et al*, 2001). This deficit of HPV18 in high-grade lesions has been observed previously (Lörincz *et al*, 1992), and it has been suggested that HPV18-associated cervical disease is more rapidly progressive, with a short duration for the HSIL lesion (Kurman *et al*, 1988). This is in agreement with our finding that the prevalence for HPV18 is highest in ASCUS and lower in LSIL and HSIL groups. Remarkable is the fact that women with LSIL infected with HPV18 are on average 5 years younger than patients with ASCUS infected with the same virus. From the above, it could be argued that LSIL is a separate end point in the disease caused by HPV infection.

Although the overall prevalence of HPV in the HSIL group (98.5%) is high, not all samples were positive. Previous studies on HPV-type prevalence in Belgium were carried out only in the female population of Antwerp on DNA extracted from paraffin-embedded tissue (Baay *et al*, 2001). Using the GP5+/6+ consensus primer they showed only a prevalence of 77% in cervical intraepithelial neoplasia (CIN), and 88% in cervical carcinoma. This could be because of the specific viral types, viral load or choice of samples. It is easier to extract HPV DNA from cytological samples than from paraffin-embedded biopsy samples. Furthermore, we found an increase in multiple infections with increasing abnormal cytology and the MY-PCR system has been shown to detect twice as much samples with multiple HPV types as compared to the GP5+/6+ PCR system (Qu *et al*, 1997). This difference in sensitivity to detect dysplasia stresses the need for validation of a consensus primer set in each given population and/or the use of simultaneous type-specific detection in some of the negative samples. The prospective comparison of the two test systems for the Belgian population will be part of a future study.

For LSIL cases the prevalence of HPV DNA is higher than 95% but still lower than in HSIL cases. The higher amount of HPV-negative LSIL cases could be explained by the fact that regression of abnormal cytology occurs 3.6 months later than HPV clearance, which may explain the presence of a lesion in the absence of HPV DNA as was shown by recent Dutch studies (Nobbenhuis *et al*, 2001; Zielinski *et al*, 2001).

We found a lower prevalence (58.6%) in ASCUS cases. This is in agreement with previous studies (Solomon *et al*, 2001; Arbyn and Van Ranst, 2002).

In this study, the prevalence of women with a normal cytology to be HPV positive was 21%, and is in agreement with other studies in which the same MY09/MY11 PCR technique was used (26.0%, Hildesheim *et al*, 1994; 17.0%, Torroella-Kouri *et al*, 1998; 25.1%, Riethmuller *et al*, 1999).

We showed that in the Flemish population HPV types 16, 18, 31, 33, 35, 39, 45, 51, 52 and 66 are present in high-grade lesions, contrary to a previous study conducted in Antwerp that detected HPV39 and 51 only in low-grade lesions (Baay *et al*, 2001). We are currently investigating differences in HPV-type prevalence in the different provinces in Flanders, including Antwerp, to see whether regional differences can explain this discrepancy.

Screening in Belgium is performed essentially at opportunistic basis, characterised by overscreening in coexistence with underscreening in indigent groups, heterogeneous quality, lack of quality control and impossibility to document effectiveness of screening by lack of adequate monitoring (Arbyn and Van Oyen, 2000).

Previous studies in the Netherlands have shown that there is a clear relation between HPV positivity and severity of disease, and that severe dyskaryosis is associated with persistent HPV infections, whereas minor abnormalities are more often associated with fluctuating or transient infections (Meijer *et al*, 1992).

HPV DNA detection allows a more effective triage of women showing equivocal cytological results than repeat cytology (Solomon *et al*, 2001; Arbyn and Van Ranst, 2002; Kim *et al*, 2002). In the case of LSIL, on the other hand, the discriminative power of HPV triage is poor because of the very high prevalence of HPV-positivity (95% in our study) (ALTS Group, 2000; Stoler, 2001). Nevertheless, HPV typing might make LSIL triage a potential useful strategy that merits further investigation.

Our study confirms that polyvalent vaccines including the main cancer-associated HPV types would be needed to prevent most cases of cervical disease in the screened, high-risk population (Herrero *et al*, 2000).

Finally, with this study we have proven that HPV typing is feasible in a high-throughput or routine setting.
